# Targeting the FSH/FSHR axis in ovarian cancer: advanced treatment using nanotechnology and immunotherapy

**DOI:** 10.3389/fendo.2024.1489767

**Published:** 2024-12-17

**Authors:** Fuqing Feng, Tianhang Liu, Xiaoman Hou, Xueyan Lin, Susu Zhou, Yongjie Tian, Xiaoyi Qi

**Affiliations:** Department of Obstetrics and Gynecology, Shandong Provincial Hospital Affiliated to Shandong First Medical University, Jinan, Shandong, China

**Keywords:** FSHR, ovarian cancer, targeted therapy, nanotechnology, immunotherapy

## Abstract

Ovarian cancer (OC) is the gynecological malignancy with the poorest prognosis. Surgery and chemotherapy are the primary therapies for OC; however, patients often experience recurrence. Given the intimate interaction between OC cells and the tumor microenvironment (TME), it is imperative to devise treatments that target both tumor cells and TME components. Recently, follicle-stimulating hormone (FSH) levels in the blood have been shown to correlate with poorer prognosis in individuals with OC. Ovarian carcinoma cells express FSH receptors (FSHRs). Thus, FSH is an important target in the development of novel therapeutic agents. Here, we review the effects of FSH on normal physiology, including the reproductive, skeletal, cardiac, and fat metabolic systems. Importantly, this review outlines the role and mechanism of the FSH/FSHR axis in the proliferation, survival, and metastasis of OC, providing theoretical support for the targeted FSHR treatment of OC. Current progress in targeting FSHR for OC, including the recent application of nanotechnology and immunotherapy, is presented. Finally, we discuss prospects and future directions of targeted FSHR therapy in OC.

## 1 Introduction

Ovarian cancer (OC) is the seventh most prevalent cancer in women worldwide, and the most lethal gynecological cancer affecting women in the United States (1). When the 5-year survival rate falls below 30%, the situation corresponds to approximately 75% of patients presenting stage III or IV disease (2). Cytopathic therapy and chemotherapy are routine treatments for patients with advanced disease (3). Although 80% of women with stage III or IV OC respond to initial treatment, the majority eventually experience relapse and develop resistance to chemotherapy. Poly (ADP-ribose) polymerase (PARP) inhibitors have emerged as potentially effective therapeutic options for individuals with wild-type BRCA epithelial OC (EOC) and BRCA mutations in recent years. Furthermore, PARP inhibitor-enriched CD133+ and CD117+ OC stem cells (OSCs) can successfully repair PARP inhibitor-induced DNA damage (4, 5). In addition, bevacizumab with platinum-paclitaxel-based chemotherapy has been suggested as the initial treatment for OC; however, although progression-free survival is prolonged by 3.5 months, no statistically significant change in overall survival (OS) is observed (6). These findings indicate that conventional treatment has reached a plateau, and that new effective treatment regimens are needed to prolong OS. At the same time, owing to the nonspecific biological distribution of chemotherapeutic agents to healthy tissues, which frequently results in serious side effects, effective therapies targeting tumor cells are needed (7) to significantly reduce systemic side effects and improve the therapeutic index of most chemotherapeutic drugs.

EOC comprises 90% of OCs and is referred to as an “immunogenic tumor” because the tumors, peripheral blood, and ascites of patients with EOC exhibit non-spontaneous antitumor immune responses (8). Ovarian tumor cells and the tumor microenvironment (TME) are closely related; thus, it is crucial to develop therapeutic strategies that can target tumor cells and retain their anticancer activity in the TME (9).

To avoid off-target interactions, identifying genes with expression limited to tumor surface targets is a significant challenge in the development of OC treatments (10). The selection of OC target sites has focused on molecules present on the surface of tumor cells, including glycosylated proteases linked to the inhibition of ovarian tumor cell invasion and metastasis (11), OC blood markers (12) (such as mucin 16 [MUC16] and human epididymis protein 4 [HE4]), and anti-angiogenic receptors (13) (such as vascular endothelial growth factor [VEGF] and epidermal growth factor receptor [EGFR]). The transmembrane tumor-associated antigen follicle-stimulating hormone (FSH) receptor (FSHR) is present in 50–70% of serous ovarian malignancies and in approximately 50% of OCs of different classes (14), but not in extragonadal tissues. According to Perales-Puchalt et al. (15), FSHR is expressed in approximately 70% of tumor microvascular endothelial cells but not in those of non-cancer-related cardiovascular origin. Therefore, targeting FSHR is the most promising strategy for overcoming OC recurrence, treatment resistance, and mortality.

Nanomaterials are a novel class of recently developed materials. Nanodrugs are often employed to create drug delivery systems that allow prolonged drug circulation and retain molecular activity of the drug while selectively targeting tumor cells to avoid the systemic side effects caused by chemotherapy medications. Immunotherapy is a recently developed field that aims to treat cancer patients by reactivating their immune systems. The US Food and Drug association has approved numerous medications of these classes for the treatment of different tumor types.

This review provides a detailed introduction to the structure and biological functions of FSH, followed by those of the FSHR, and summarizes the mechanism of action of FSH and FSHR in the occurrence and development of OC. The research progress and future development prospects of nanotechnology and immunotherapy for targeting FSHR in OC are highlighted, aiming to provide further theoretical evidence supporting targeted therapy of OC.

## 2 FSH

The anterior pituitary gland produces FSH, a pituitary gonadotropin involved in the regulation of gonadal functions (16). Gonadotropins belong to a family of closely related glycoproteins, including FSH, thyrotropin (TSH), chorionic gonadotropin (CG), and luteinizing hormone (LH). These hormones are produced by various cell types: pituitary cells synthesize TSH, gonadal cells synthesize LH and FSH, and placental trophoblasts produce CG (17). Every member of the family has a structurally unique beta (β)- component in addition to a functionally indispensable 96-amino acid alpha (α)-subunit shared by LH, TSH, and CG. FSH is a heterodimer consisting of a non-covalently bound common alpha subunit to a unique beta subunit that confers biological specificity to the hormone (18). The individual subunits have no known biological activity. Nonetheless, heterodimer formation is essential for this activity. In silico and crystallographic structural investigations revealed an interaction between the α subunit and the FSHR, indicating that receptor binding is not limited to the β subunit (19). Biological functions of FSH are activated by its interaction with FSHR; thus, the FSHR plays a central role in human reproduction.

## 3 FSHR

Encoded by 11 exons and 10 introns, the human FSHR gene is located on chromosome 2 and spans 52 kb. The FSHR protein contains 695 amino acids, including a 17-amino acid signal peptide sequence, whereas the mature receptor contains 678 amino acids and 3–4 potential glycosylation sites (20). In sheep and mouse gonadal tissues, pre-mRNAs encoding this gene undergo alternative splicing, creating several subtypes (FSHR1, FSHR2, FSHR3, and FSHR4) (21). FSHR1 is also referred to as the 7-trans-membrane receptor (7TMR) due to its seven transmembrane helices. It comprises an N-terminal extracellular domain, seven transmembrane domains, three extracellular loops, three intracellular loops, and a C-terminal intracellular domain. The glycosylated extracellular domain consists of hormone-binding and signal-specific subdomains and has 12 leucine-rich repeats. Hormonal activity is caused by a sulfated tyrosine residue at the hinge loop position 335 in the signal-specificity subdomain (22).

FSHR1: Sertoli and granulosa cells express the mature FSHR1 protein, which has a molecular mass of 76 kDa (23) and is involved in follicular development, differentiation, and proliferation, and hormone production in granulosa cells (24). It consists of an intracellular C-terminal tail, seven α-helical transmembrane domains connected by alternating extracellular and intracellular loops, and a sizable N-terminal extracellular domain (25). Preclinical investigations have shown that the FSH/FSHR1 pathway is associated with increased angiogenic potential of ovarian granulosa cells and is beneficial for follicular maturation. The FSH/FSHR1 complex appears to enhance the secretion of platelet-derived growth factor receptor-β (PDGF-β) and VEGF in granulosa cells mediated by transforming growth factor-β1 (TGF-β1). The FSH-FSHR1 interaction is involved in the vascular development of this rapidly growing cell population (26). By binding to intracellular C-terminal fragments and FSH, FSHR-1 initiates several intracellular signaling cascades. Of these, cyclic adenosine monophosphate (cAMP)/Protein kinase A (PKA) signaling is the most prevalent (27).

FSHR2: FSHR2 consists of 10 exons but a different C-terminus (28). In contrast to FSHR1, FSHR2 preserves the extracellular and transmembrane domains of the receptor but not the intracellular domains, and this truncation negatively affects downstream signaling processes. Although FSHR2 binds to FSH with high affinity, it cannot induce G protein-coupled receptor signaling; therefore, it is the dominant inactivated receptor (29, 30). This dominant-negative FSH receptor may be involved in an inhibitory pathway and likely activates Gi after binding to FSH (31). However, the biological functions of FSHR2 have not been reported.

FSHR3: FSHR3, also known as the growth factor 1 receptor, contains exons 1–8, but is a truncated protein (28) with a single transmembrane segment, and lacks the hepta-helical R1 region implicated in coupling with heteromeric G proteins. It is a typical receptor for cytokines/growth factors (21). Feature-wise, FSHR3 is distinct from FSHR1. It operates apart from cAMP-regulated pathways (32). The C-terminus of FSHR3 contains the PVILSP sequence, which may be a common motif for MAPK phosphorylation (21, 33). FSHR3 activates the mitogen-activated protein kinase-extracellular signal-regulated kinase (MAPK/ERK) pathway in granulosa cells in a cAMP-independent manner (31, 32, 34). FSHR3-mediated activation of the MAPK/ERK pathway regulates cell proliferation through calcium ion (Ca2+) influx by modulating Ca2+-dependent channels (33, 35). The proliferation of ovarian epithelial cells in response to FSHR3-mediated MAPK activation further demonstrates its role in promoting mitosis and proliferation (35). FSH acts directly on endogenous tissue stem/progenitor cells in the gonads and bone marrow via FSHR3, promoting asymmetrical and symmetrical cell division and clonal expansion (36).

FSHR4: FSHR4 contains only exons 1–4 and is referred to as the soluble FSHR because it lacks a transmembrane domain (28). FSHR4 stabilizes or inhibits the binding of FSHR to FSH in the extracellular matrix. FSHR4 also functions as an insulin-like growth factor (IGF-1)-binding protein (37) and has been suggested to be a prohormone of active molecules (38). Expression of several FSHR variants in cells may be linked to multiple FSH signal transduction pathways (39).

## 4 FSH recognizes and activates the FSHR signaling pathway

FSHR mediates its biological role in target cells through the stimulatory Gs alpha subunit (Gαs)/cAMP/PKA signaling pathway (40, 41). Interestingly, evidence suggests that the FSH/FSHR system functions through other pathways.

### 4.1 Canonical Gαs/cAMP/PKA pathway in target cell FSHR

The typical Gαs pathway indicates that the FSH-FSHR interaction leads to receptor coupling with Gαs subunits, inducing adenylate cyclase activation and cAMP production (42). In turn, cAMP activates downstream effector protein kinase A (PKA). FSH-mediated activation of the cytosolic ERK/MAPK pathway is PKA-dependent. In addition, FSH-induced MAPK p38 phosphorylation is PKA-dependent.

### 4.2 PI3K/mTOR signaling at FSHR in target cells

The PI3K/mTOR signaling pathway plays a crucial role in FSH-induced cell proliferation, gene expression, and protein translation. Multiple studies have confirmed that the PI3K/mTOR pathway serves as an effector pathway for FSH/FSHR-mediated signaling. FSH acts on the FSHR receptor to activate PI3K, which promotes the conversion of phosphatidylinositol 4,5-bisphosphate(PIP2) to (phosphatidylinositol-3,4,5-triphosphate)PIP3 (41) and activates Akt (43), which subsequently activates downstream signaling cascades, including the phosphorylation and inactivation of GSK3β (44) and AMPK (17), as well as the inactivation of transcription factors Fox3a and FoxO1 (41).

### 4.3 FSH-induced β-arrestin-dependent pathway

The β-arrestin pathway, initially serving as a pathway for FSHR desensitization and recycling, has gradually gained recognition as an adapter and converter for FSH/FSHR signaling (45). β-arrestin-mediated receptor recycling initiates G protein-independent signal transduction, not only through FSHR but also through several other 7TMRs (46). Interestingly, unlike the rapid and transient nature of G protein-mediated signal transduction, the signaling cascade involving the β-arrestin pathway is gradual and sustained (47).

### 4.4 FSH-mediated nuclear signaling pathway

FSH regulates the expression of genes involved in steroidogenesis (48). Upon stimulation, multiple signaling pathways (PKA, p38, ERK, and Akt) (43, 49, 50) are activated, which subsequently trigger the activation or inhibition of different transcription factors in the nucleus through nuclear translocation, thereby regulating gene expression.

### 4.5 Proteins interact with the FSHR, influencing signaling pathways

The above signaling pathways are primarily activated by second messengers Furthermore, FSHR interacts directly with specific proteins to regulate signaling cascades via alternate pathways. The proteins 14-3-3τ, APPL1, and FoxO1a directly act on the FSHR, with the latter two controlling the downstream PI3K/Akt pathway of FSHR, thereby modulating gene expression (51, 52).

## 5 FSH functions through the FSHR

### 5.1 Role of FSH in reproduction

**Figure 1:**
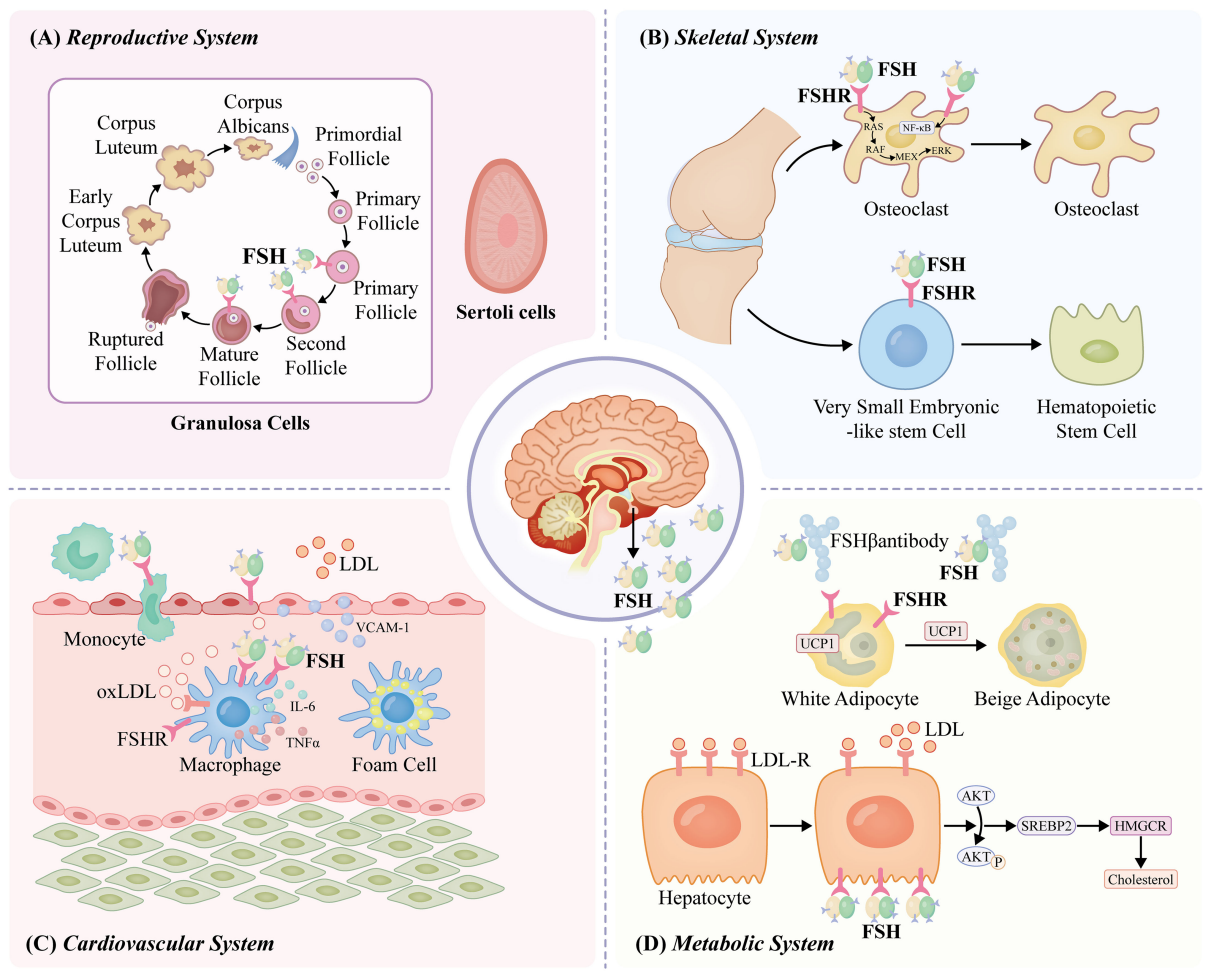
FSH functions through the FSHR. In females, FSH plays a key role during ovarian folliculogenesis and antral follicle development and, in combination with luteinizing hormone (LH), stimulates preovulatory follicular growth (56–58). In males, FSH regulates the mitotic proliferation of Sertoli cells, supports their growth and maturation, and prompts the release of androgen-binding protein, which regulates the overall process of spermatogenesis (60). FSH acts on FSHRs on osteoclasts, stimulating NFκB, MEK/Erk, and AKT pathways, thus promoting osteoclast formation, function, and survival (63, 64). Functional FSHR is expressed on bone marrow very small embryonic-like stem cells (VSELs) and hematopoietic stem cells (HSCs) in adult mice (65). PKA further phosphorylates a large number of cytokines and regulates cAMP-response element binding protein (CREB) transcription in the nucleus to control the expression of downstream effector genes, such as UPC1, aromatase, and inhibin-A (66). FSH interacts with FSHRs in HepG2 cells, reducing LDLR levels (67). Mature SREBP2 increases liver cholesterol synthesis by promoting transcription and expression of the cholesterol synthesizing and rate-limiting enzyme 3-hydroxy-3-methylglutaryl coenzyme A reductase (HMGCR) (68). The interaction of FSH with FSHR on monocytes has been shown to upregulate RANK expression and promote monocytic infiltration of atherosclerotic plaques (69). FSH may act by stimulating new vessel formation via FSHR present on vascular endothelial cells (70). FSH may elevate the production of cytokines, namely IL-6 and TNFα, from macrophages to cause low-grade inflammation, atherosclerosis development, and insulin resistance (71). FSH promotes the development of atherosclerosis by increasing VCAM-1 protein expression via activating the PI3K/Akt/NF-κB pathway (72).

FSH and LH are released in response to the pulsatile release of the gonadotropin-releasing hormone (GnRH). Estradiol and inhibin B are the two primary factors that suppress FSH secretion (53–55). FSH secretion and activity have been linked to different pituitary regulatory proteins, including follistatin and activin (54). FSH is essential for the development and control of the male and female reproductive systems because it acts on the FSHR, which is mostly expressed in granulosa and Sertoli cells (56). In females, FSH is essential for ovarian folliculogenesis and antral follicle formation, and along with LH, it promotes preovulatory follicular growth (56–58). Additionally, OSCs express FSH and respond to FSH/FSHR signaling, resulting in self-renewal, clonal amplification, new ovum formation, and primordial follicle assembly (59). FSH controls the growth and maturation of Sertoli cells in males, promotes mitotic proliferation, and triggers the release of androgen-binding proteins that control spermatogenesis (60). Notably, this study indicates that inadequate FSHR signaling through the placental endothelium has a negative impact on the growth of both the fetus and the placenta, and that endothelial-cell FSHR in the placental vasculature promotes angiogenesis (61, 62). The role of FSHR may have previously been overlooked in both the remodeling of the mother’s spiral arteries and embryo implantation. However, further investigations are required (**Figure 1**).

### 5.2 Effects of FSH on the bone and bone marrow

In perimenopausal or postmenopausal women, decreased estrogen production due to ovarian senescence has historically been the primary cause of bone loss. Estrogen replacement therapy is thought to be a reasonable therapeutic option in this population (73). However, perimenopausal bone loss is not only reliant on estrogen; the impact of FSH on the bone may also play a role. In women of reproductive and non-reproductive ages, as well as those undergoing menopausal transition, FSH levels have been linked to bone loss (67, 74). Women with higher estrogen-to-FSH ratios showed less bone loss in the lumbar spine during perimenopause (75). Genetic research has demonstrated that regardless of FSH and estrogen levels, women with an active FSHRN680S polymorphism have a greater risk of developing postmenopausal osteoporosis (76).

By activating the nuclear factor kappa B(NFκB), MEK/Erk, and AKT pathways in response to FSH acting on FSHRs on osteoclasts, osteoclast production, function, and survival are enhanced (63, 64). In addition, FSH stimulates osteoclasts through an indirect pathway: interleukin (IL)-1β, IL-6, and tumor necrosis factor-alpha (TNFα) production are increased in proportion to FSHR expression due to the overexpression of receptor activator NFκB (RANK) (77, 78). Furthermore, FSH promotes osteoclastogenesis by interacting with the immunoreceptor tyrosine-based activation motif (ITAM) adaptor (79). Amber et al. (65) reported the expression of functional FSHR in the bone marrow of very small embryonic-like stem cells (VSELs) and hematopoietic stem cells (HSCs) in adult mice. VSELs are the most primitive pluripotent stem cells in the bone marrow and are capable of self-renewal and HSC production under 5-fluorouracil (5-FU)- and FSH-stress. HSCs further divide and differentiate to maintain balanced levels in the body. Functional studies by our group on the bone marrow of adult mice have shown a direct effect of FSH on hematopoiesis (65). In summary, accumulating evidence has shown that FSH acts directly on the bone and bone marrow through a specific FSHR, which increases osteoclastogenesis, stimulates bone resorption, and promotes hematopoiesis.

### 5.3 Effects of FSH on fat metabolism

Compelling evidence indicates that human, mouse, and chicken adipocytes express the transcription factor FSHR (56). Pleiotropic FSHR signaling promotes FSH, which, in turn, controls fat metabolism. PKA, cAMP, and intracellular adenylate cyclase (AC) are traditionally activated by Gαs that is induced by FSH binding to FSHR. PKA also controls the transfection of cAMP-response element-binding protein (CREB) in the nucleus, which phosphorylates many cytokines and regulates downstream effector genes such as inhibin-A, aromatase, and uncoupling protein 1 (UCP1) (66). GnRH stimulation increases FSH levels and cyclin D1 (CCND1) and cyclin E1 (CCNE1) transcription via the PKA/CREB pathway (80). Adipocyte fat accumulation is promoted by CCND1 and CCNE1, which drive cell cycle progression and enhance cell differentiation. Furthermore, FSH directly stimulates 3T3-L1 cells and primary murine adipocytes via the inhibitory G α subunit (Gαi)-coupled FSHR, which results in the upregulation of key genes involved in lipid metabolism, such as Fas, Lpl, and Pparg, as well as the stimulation of lipid synthesis (56). The bone marrow and subcutaneous fat were significantly reduced in different rat models following injection of an anti-FSHβ antibody. These models included ovariectomized mice and mice that were either pair-fed a high-fat diet or allowed unrestricted access to standard chow (81).

FSH also inhibits hepatic cholesterol metabolism. In HepG2 cells, FSH interacts with FSHRs to lower LDLR levels (82). Reduced LDLR expression inhibits LDL-C endocytosis and increases circulating LDL levels. Furthermore, β-arrestin 2 modulates the formation of the FSH/FSHR complex, which activates Gi2α and increases Akt activation via PI3K. The sterol regulatory element-binding protein 2 (SREBP2) transcription site is released, and FOXO1 nuclear transfer is inhibited by phosphorylated Akt, which increases SREBP2 transcription and expression. By encouraging the transcription and expression of cholesterol, which produces the rate-limiting enzyme 3-hydroxy-3-methylglutaryl coenzyme A reductase (HMGCR), mature SREBP2 promotes hepatic cholesterol production (68).

### 5.4 Effects of FSH on the cardiovascular system

Multiple studies have indicated that FSH levels are associated with cardiovascular diseases. Whether and to what extent FSH levels determine the rate of cardiovascular occurrence remains controversial. Men receiving androgen-deprivation therapy (ADT) for prostate cancer are more susceptible to thrombosis, atherosclerosis, and cardiovascular failure (83–85). For example, FSH was recently demonstrated to increase the risk of cardiovascular disease in males undergoing ADT (69). In addition, a positive connection was observed between the FSH trajectory and intima-media thickness in the SWAN trial, which involved 856 women who did not report having a stroke or heart attack (86). However, a 22-site population-based investigation on the frequency of metabolic disease-related risk factors in East China revealed a negative relationship between FSH levels and cardiovascular risk (87). The interplay between monocyte FSH and FSHR stimulates RANK production and promotes infiltration of monocytic cells into atherosclerotic plaques (69). Furthermore, as neovascularization is necessary for the development of atherosclerotic plaques (88), FSH may promote the production of new vessels through FSHR in vascular endothelial cells (70). FSH can enhance macrophage cytokine production, including IL-6 and TNFα, leading to low-grade inflammation, atherosclerosis, and insulin resistance (71). Additionally, FSH contributes to the development of atherosclerosis by activating the PI3K/Akt/NF-κB pathway and boosting vascular cell adhesion molecule-1(VCAM-1 )protein expression (72). Collectively, these factors play significant roles in the onset and progression of atherosclerosis. However, the specific mechanism of action of FSH/FSHR in the development of atherosclerosis requires further investigation.

### 5.5 Role of FSH-FSHR in OC

**Figure 2:**
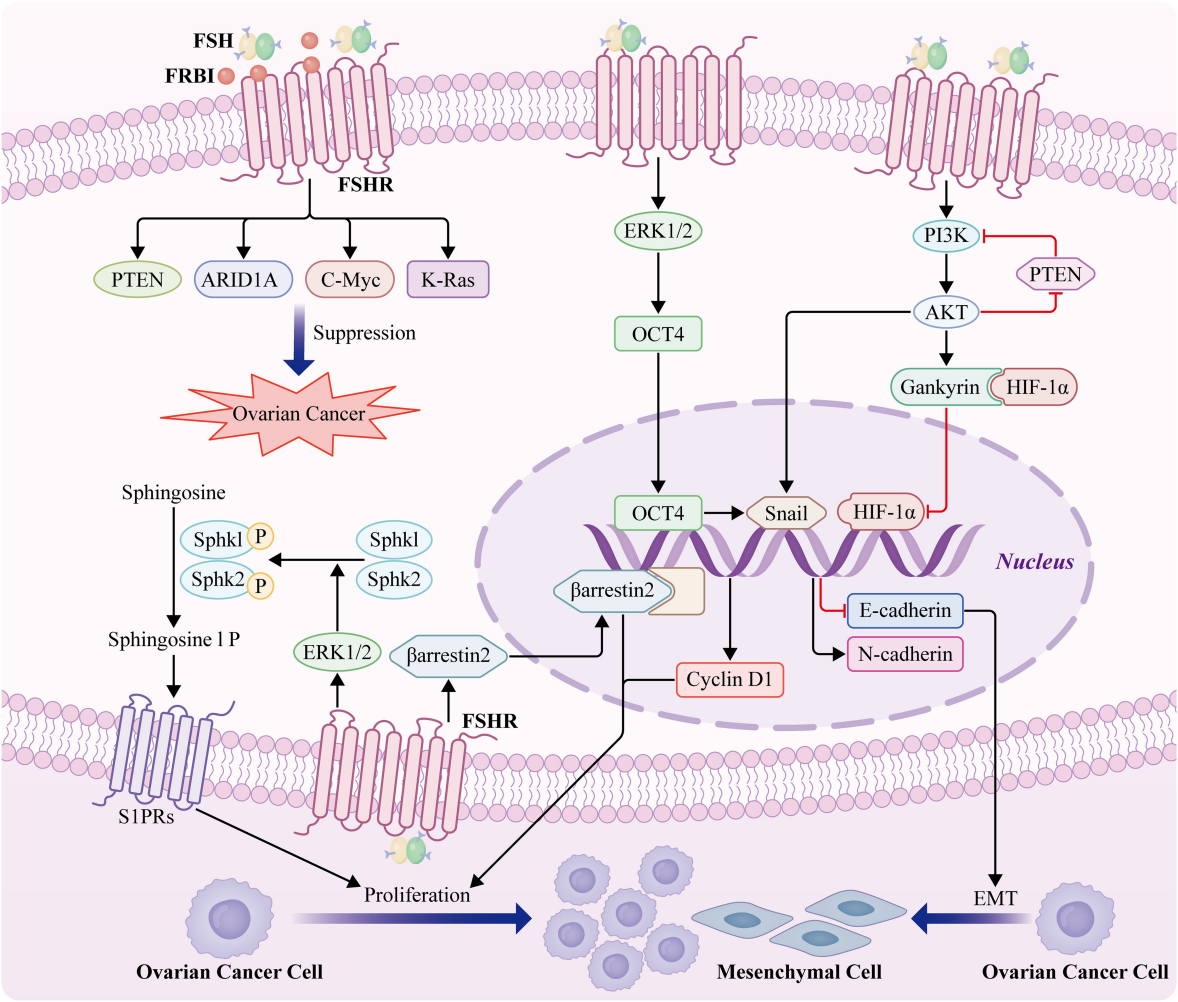
Mechanism of action of FSH-FSHR in OC proliferation and EMT. Cyclin D1 expression is induced by gankyrin-mediated HIF-1α via a gankyrin-established PI3K/AKT signaling feedback loop (91). FSH stimulates the phosphorylation of both sphingosine kinase1 and sphingosine kinase2 and thus regulates the survival and growth of OC cells by activating Sphk1 and Sphk2 through ERK1/2 (92). High levels of β-arrestin 2 are positively correlated with the expression of the gonadotropin receptors FSHR. β-arrestin 2 expression significantly facilitates the proliferation of OC cells (93). FSH induces epithelial OC invasion via the regulation of the ERK1/2 signaling pathway, resulting in the upregulation of OCT4 expression and subsequently, EMT and invasion (94). FSH-induces EMT of epithelial OC cells through FSHR PI3K/Akt-Snail signaling pathway (95). FSH receptor binding inhibitor (FRBI), as an FSH antagonist, blocks FSH binding to FSHR8 and changes the role of FSH at the receptor level. FSH receptor binding inhibitor upregulates ARID1A and PTEN genes associated with OCs in mice (96). A high dose of FRBI reduces the production of c-Myc and K-Ras, which probably prevents or blocks tumorigenesis and progression of OC (10).

Patients with OC have elevated FSH levels. Previous studies have reported higher FSH levels in OC and gynecological malignancies (89); however, the relationship between high FSHR expression, progression-free survival (PFS), and overall survival (OS) remains controversial. Furthermore, increased levels of putative carcinogenic pathways and an increase in the number of preneoplastic ovarian surface epithelial cells may be associated with FSHR overexpression (90). Endothelial FSHR expression correlates with vascular remodeling and angiogenesis. However, the mechanisms underlying FSH-driven signaling pathways that lead to EOC development remain unclear. Therefore, we have summarized the mechanism by which FSH-FSHR promotes OC cell proliferation and epithelial-mesenchymal transition(EMT) (**Figure 2**).

It has been proposed that the growth factor receptor FSHR3, which triggers the MAPK/ERK pathway in a Ca2+-dependent manner, is the mechanism by which FSH promotes OC cell growth (35). Overexpression of FSHR-3 is also associated with increased cell proliferation and aggressive behavior, because it promotes the expression of epidermal growth factor receptor 2 (EGFR-2), EGFR, and c-Myc (97). Furthermore, Gankyrin mediates FSH-driven OC cell proliferation by regulating the stability of HIF-1α protein and the expression of cyclin D1 (91). Additionally, our study showed that FSH stimulates the phosphorylation of both sphingosine kinase 1 (Sphk1) and sphingosine kinase 2 (Sphk2) and regulates the survival and growth of OC cells by activating Sphk1 and Sphk2 through ERK1/2 (92). To our knowledge, this is the first study to provide evidence that high levels of phospho-SphK1 and phospho-SphK2 are prognostic indicators of OS in patients with EOC. Another group reported that high levels of β-arrestin 2 were positively correlated with the expression of the gonadotropin receptor, FSHR. Meanwhile, this study revealed that β-arrestin 2 expression significantly facilitates the proliferation of OC cells (93). Importantly, spheroids in the ascites fluid express and secrete FSH, which regulates cancer cell proliferation and spheroidogenesis through Notch signaling, suggesting that FSH is an autocrine regulator of cancer metastasis. FSH plays an important role in OC cell proliferation. A recent study demonstrated that FSH induces EOC invasion by regulating the ERK1/2 signaling pathway, resulting in the upregulation of octamer-binding transcription factor 4 (OCT4), and subsequent EMT and invasion (94). Additionally, FSH-induced EMT in EOC cells may occur via the FSHR PI3K/Akt-Snail signaling pathway (95). Taken together, these studies demonstrated that FSHR activates oncogenic pathways and facilitates an invasive phenotype, even in the absence of FSH.

Cancer stem cells drive cancer progression by regulating signaling pathways and miRNAs, thereby providing resistance to treatment and promoting metastasis (98). FSH plays an important role in promoting cancer stem cells in malignant tumors. FSH exhibits anti-apoptotic effects in OSC by regulating stem cell signaling pathways (99). OCT-4 is a stem cell marker that is overexpressed in several types of human cancers and can induce chemotherapy resistance and inhibit apoptosis. FSH/FSHR upregulates the expression of OCT-4 in OC stem cells, and OCT-4 overexpression increases Notch, Sox 2, and NANOG levels, resulting in the expansion of CD44+/CD117+ cells that exhibit stem cell characteristics (99).

Regarding relationships between FSHR polymorphisms and OC, the National Center for Biotechnology Information (NCBI) single nucleotide polymorphism (SNP) database has identified 731 SNPs in FSHR genes, some of which are associated with cancer susceptibility (100). Yang et al. (101) reported an association between FSHR subtypes and OC in an Asian population, but no similar results were observed in a Caucasian population. Greb et al. (102)found an association between the Ser/Ser genotype of FSH and a prolonged menstrual cycle (101). Therefore, we speculated that the FSHR Asn680Ser polymorphism is associated with cancer susceptibility.

## 6 Advances in targeting FSHR as a treatment for OC

**Figure 3:**
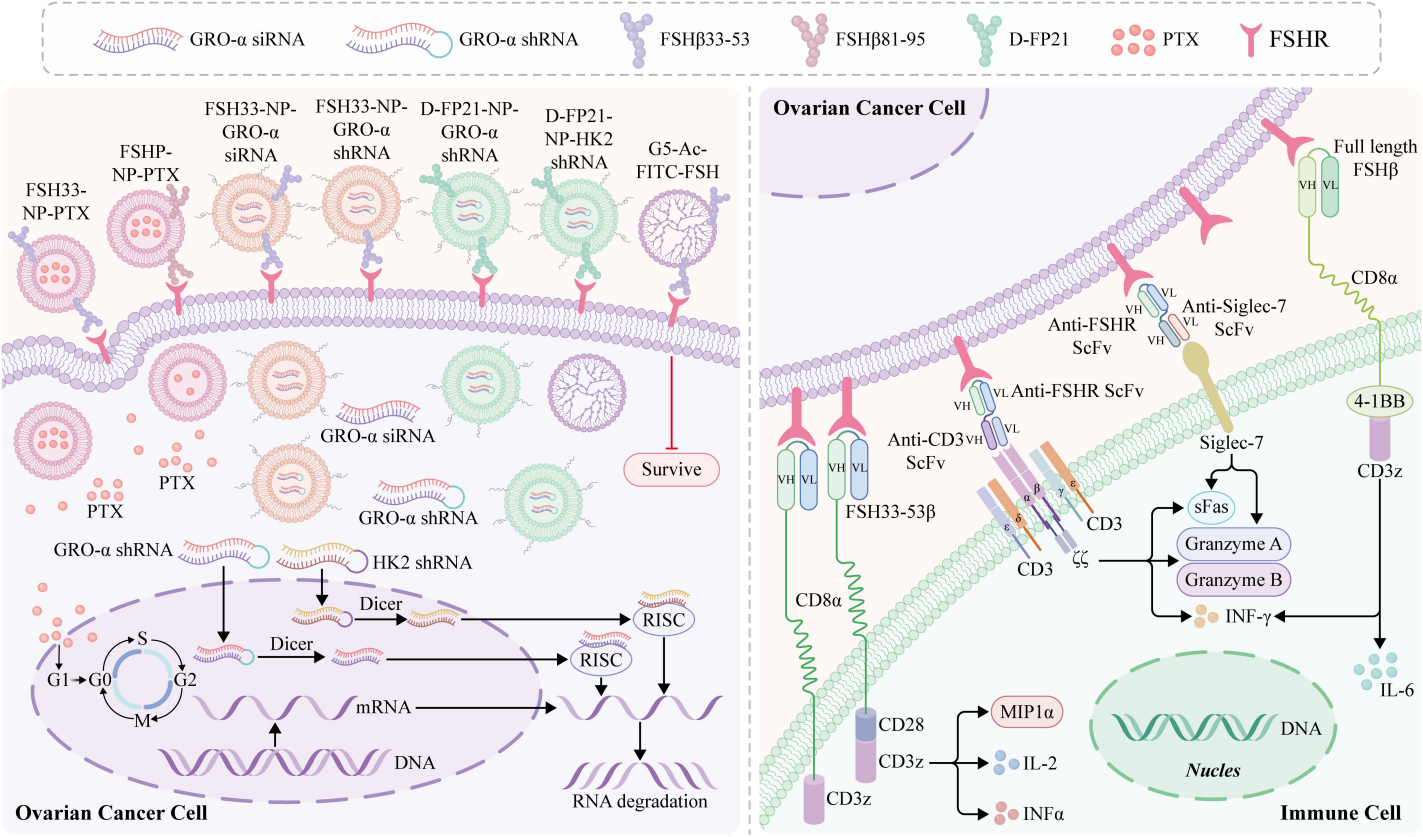
Nanotechnology and immunotherapy for ovarian cancer (OC) targeting FSHR. Follicle-stimulating hormone peptide (FSHP) facilitates paclitaxel nanoparticles’ (NPs) targeting of ovarian carcinoma *in vivo* (14). The FSHP-NP-PTX system recognizes metastasic lymph nodes of ovarian cancer and can be captured by the lymph nodes by FSHP-NP-PTX passively targeting the lymphatic system, thereby significantly inhibiting cell proliferation (105). An siRNA-targeted NP delivery system with follicle-stimulating hormone (FSH)β 33-53 peptide as the targeting ligand inhibits the migration and invasion of ovarian clear cell cancer cells, which is an effective targeted therapy strategy for ovarian cancer and a stable delivery system for siRNA (106). FSH peptide-conjugated NPs with an increased amount of polyethylene glycol (PEG) grafting and encapsulated short hairpin RNA (shRNA) can silence the FSH target gene, growth-regulated oncogene α (gro-α) (107). Moli et al. (108) designed a novel FSH 33-targeting dendritic macromolecular nanocarrier as a potential delivery platform for OC cells that express FSHR, which is a highly effective active targeting medium and has the potential to block the FSH signaling pathway cascade while selectively delivering chemotherapy drugs, potentially enhancing its therapeutic effect. Preparation of ovarian cancer FSHR NP vector carrying therapeutic plasmid growth-regulating oncogene α (pGRO-a) short hairpin RNA (shRNA) (FP21-PEG-PEI/pGRO-a). A strategy to treat human ovarian cancer by redirecting primary human T cells to target FSHR laying a foundation for further development of FSHR-targeted immunotherapy (109). In addition, T-cells redirected against FSHR+ tumor cells with full-length FSH represent a promising therapeutic alternative against a broad range of ovarian malignancies, with negligible toxicity even in the presence of cognate targets in tumor-free ovaries (89). mAb targeting the external domain of FSHR using an *in vivo*-expressed FSHR vector and identifying an effective surface targeting mAb D2 AP 11 (10). DB7.2xD2AP11 DNA-encoded bispecific NK cell engager exhibits *in vitro* expression and binding to Siglec-7 and FSHR, which induces potent killing in multiple ovarian tumor lines and decreases tumor burden *in vivo* (110).

Targeted therapies have been developed over several years along with an extensive understanding of the molecular and genetic changes in cancer. The potential use of FSHR as a therapeutic target has also been investigated. One study examined the effect of sunitinib treatment on FSHR expression in kidney cancer cells (103). Digarek, a GnRH-receptor antagonist, was used to block FSH production in a patient with metastatic colon cancer who had received multiple treatments, with encouraging results (104). Nanotechnology and immunotherapy targeting FSHR therapy for OC have brought new prospects for the treatment of OC (**Figure 3**).

### 6.1 Nanotechnology for targeted FSHR treatment of OC

Recent studies on OC have focused on drug carrier-based targeted therapies. Nanotechnology plays an important role in OC diagnosis and treatment. Owing to the unique characteristics of matter at the nanoscale, nanomedicine has emerged as a potent tool for creating novel drug carriers that are more effective and have fewer adverse effects than traditional treatments. Nanocarriers can assist in the targeted delivery of hydrophobic compounds, stabilize delivery carriers, reduce systemic toxicity of antineoplastic agents, and enhance the biodistribution and pharmacokinetics of active pharmaceutical ingredients (111). Nanocarriers, including liposomes, dendrimers, polymer nanoparticles (NPs), and polymer micelles, possess unique surface chemistries, morphologies, and mechanisms of action that can be exploited to distinguish between malignant and normal cells, thus paving the way for targeted drug delivery. Furthermore, the development of specific ligand-functionalized nanoformulations enables preferential targeting of ovarian tumors and eventually amplifies their therapeutic potential compared with their non-functionalized counterparts (112).

Several functionalized nanoparticles(NPs) have been tested for use in cancer diagnosis and treatment. However, clinical translation remains limited, and NP-based health applications continue to face challenges (113). According to a recent survey, the success rate of phase I trials involving nanomedicines was approximately 94%, that of phase II trials was as low as 53%, and that of phase III trials dropped further to 18% (114). The reason for the failure of clinical conversion rate is its low efficacy, which may be attributed to biological barriers, NP toxicity, physical and chemical characteristics, scale effects, and limitations of animal models (115). Below, we focus on the obstacles that reduce the effective accumulation of NPs at tumor sites and their targeted delivery. Despite recent advances, the toxicity of nanomaterials and biological barriers remain significant obstacles.

Biological barriers: After intravenous injection, nanomedicines undergo a complex multistep cascade process in the body to exert their efficacy, which includes entry into the blood circulation, accumulation at the tumor site, penetration into the interior of tumor tissue, endocytosis, intracellular transport, and drug release. A low efficiency at any step reduces the overall delivery effect (116). Numerous proteins in the blood can tightly bind to form a “protein crown” on the NPs surface, thus changing their physicochemical characteristics and stability, hindering the specific binding of targeted molecules to receptors (117). Most NPs are taken up and cleared by macrophages or endothelial cells in the liver or spleen after entering the body, hindering further delivery to tumor tissues (118). In addition, the “protein crown” can trigger immune responses, resulting in the clearance of NPs from the body before reaching the target site. The extravasation of NPs into tumors through the blood circulation is affected by tumor vascular distortion, the surrounding microenvironment, and the properties of NPs (119). The enhanced permeability and retention (EPR) effect leads to the accumulation of NPs, which has become the “gold standard” for designing tumor-targeted delivery systems (120). However, the EPR is effective in animal models but is usually ineffective in patients (121). Most importantly, the EPR effect not only varies greatly in various mouse tumor models with different tumor vascular pathophysiological characteristics, but also varies greatly among patients owing to inherent tumor heterogeneity and individual factors (122). Therefore, innovative tools, technologies, and strategies must be adopted to effectively solve these problems.

NP toxicity: NPs significantly alter the *in vivo* distribution characteristics of drugs, accumulating more in normal tissues, such as in the liver and spleen. Clinical research failures owing to toxicity are common (123). Furthermore, these negative impacts are determined by the material properties of the NPs. For example, metallic NPs can cause neurotoxicity by inducing ROS generation and cytokines. This facilitates neuroinflammation, impairs synaptic transmission, and leads to brain cell death (124). Therefore, overcoming the safety issues caused by the distribution characteristics is a prerequisite for the widespread application of nanomedicine.

Tumor cell-specific targeting is an effective strategy to enhance the internalization and therapeutic efficacy of nanomedicine. After functionalization with targeted ligands such as antibodies, aptamers, peptides, and small molecules, nanomedicines can specifically recognize and bind to receptors or other molecules on the surface of tumor cells, leading to increased retention of tumor tissue and enhanced uptake by tumor cells.

Much of the current literature on OC has focused on FSHR-based targeted therapies. As a specific receptor, FSHR has significant advantages in cancer cell targeting. This receptor is endocytosed by cells and is usually expressed more in cancerous cells than in healthy cells. The internalization and varied expression of this receptor allow for the reduction of unfavorable side effects and enhancement of therapeutic outcomes. Therefore, the development of improved treatments using enhanced delivery systems is a primary goal of NP-therapy. Follicle-stimulating hormone peptide (FSHP) can facilitate paclitaxel (PTX) NPs targeting ovarian carcinomas *in vivo* (14). This novel FSH33-NP-PTX displays stronger antiproliferative and antitumor effects than free PTX or naked PTX-loaded NPs (NP-PTX), both *in vitro* and *in vivo*, and not only enhances the antitumor effect of chemotherapeutic drugs, but also minimizes side effects in unrelated normal organs. Furthermore, FSHP-NP-PTX recognizes and accumulates in metastatic lymph nodes in OC by passively targeting the lymphatic system, which can significantly inhibit cell proliferation (105). Previously used chemotherapeutic drugs, such as paclitaxel, target OC cells themselves rather than the entire tumor tissue. The stroma surrounding cancer epithelial cells not only provides a supportive and nutritive microenvironment for cancer cells but also assists in the development and progression of cancer. A different study used an siRNA-targeted NP delivery system to target FSH β 33–53 peptide and effectively inhibited the migration and invasion of ovarian clear cell cancer cells, indicating NPs can be used an effective targeted therapeutic strategy for OC and a stable delivery system for siRNA (106).

Another limitation of NP-based medicines is the acute toxicity observed *in vivo*, which may be due to a small amount of polyethylene glycol (PEG) grafting, which leads to polyplex aggregation. An FSH peptide-conjugated NPs was developed with increased PEG grafting that encapsulated short hairpin RNA (shRNA) to silence the target gene growth-regulated oncogene α (Gro-α) (107). Gro-α shRNA-loaded NPs conjugated with FSH peptides overcome the drawbacks of the *in vivo* application of RNAi therapeutics and polymer-based nanocarriers and show safe antitumor efficacy. Previously, Moli et al. (108) designed a novel FSH 33-targeting dendritic macromolecular nanocarrier as a potential delivery platform for OC cells expressing FSHR, which was a highly effective active targeting medium and had the potential to block the FSH signaling pathway cascade while selectively delivering chemotherapeutic drugs, and potentially enhanced its therapeutic effect. Zhang et al. (125) conjugated a 21-amino acid polypeptide (L-FP21) with polyethyleneimine (PEI) and methoxy PEG (MPEG). An OC FSHR NP vector was prepared carrying therapeutic plasmid, growth-regulating oncogene α (pGro-α) shRNA (FP21-PEG-PEI/pGro-a). In another recent study, HK2 shRNA-loaded NPs were developed with FSH β 33–53 or retro-inverso FSH β 33–53 peptide modification to target tumor metabolism and growth in OC, which effectively suppressed HK2 expression, reversed glycolytic-based glucose metabolism and exhibited strong antitumor effects even in cisplatin-resistant OC with negligible systemic toxicity (126). Multiple studies have indicated that nanomaterials targeting FSHR have tremendous potential for the development of novel therapeutic drugs for the treatment of aggressive cancers, such as EOC, thereby opening up new opportunities in cancer research.

### 6.2 Immunotherapy for targeted FSHR treatment of OC

With recent improvements in our understanding of the molecular basis of tumor immune recognition and regulation, immunotherapies have attracted great interest. These include immune checkpoint inhibitors (ICI), cancer vaccines, and adoptive cell therapies (ACT) (6). Despite its strong rationale, immune checkpoint blockade using antibodies targeting programmed cell death 1 (PD-1) or programmed cell death ligand 1 (PD-L1) has shown limited efficacy in EOC treatment with a modest response rate of 4–15% (127). This can be partly explained by the fraction of immunologically uncold ovarian tumors and the comparatively modest number of tumor-resident tumor-reactive effector T cells that are crucial for antitumor activity and therapeutic response (128, 129). Further research is needed to understand the mechanism of immune resistance in OC, including the immunological, genetic, and molecular aspects, which are crucial for developing effective immunotherapies for patients with OC and improving their clinical outcomes.

**Figure 4:**
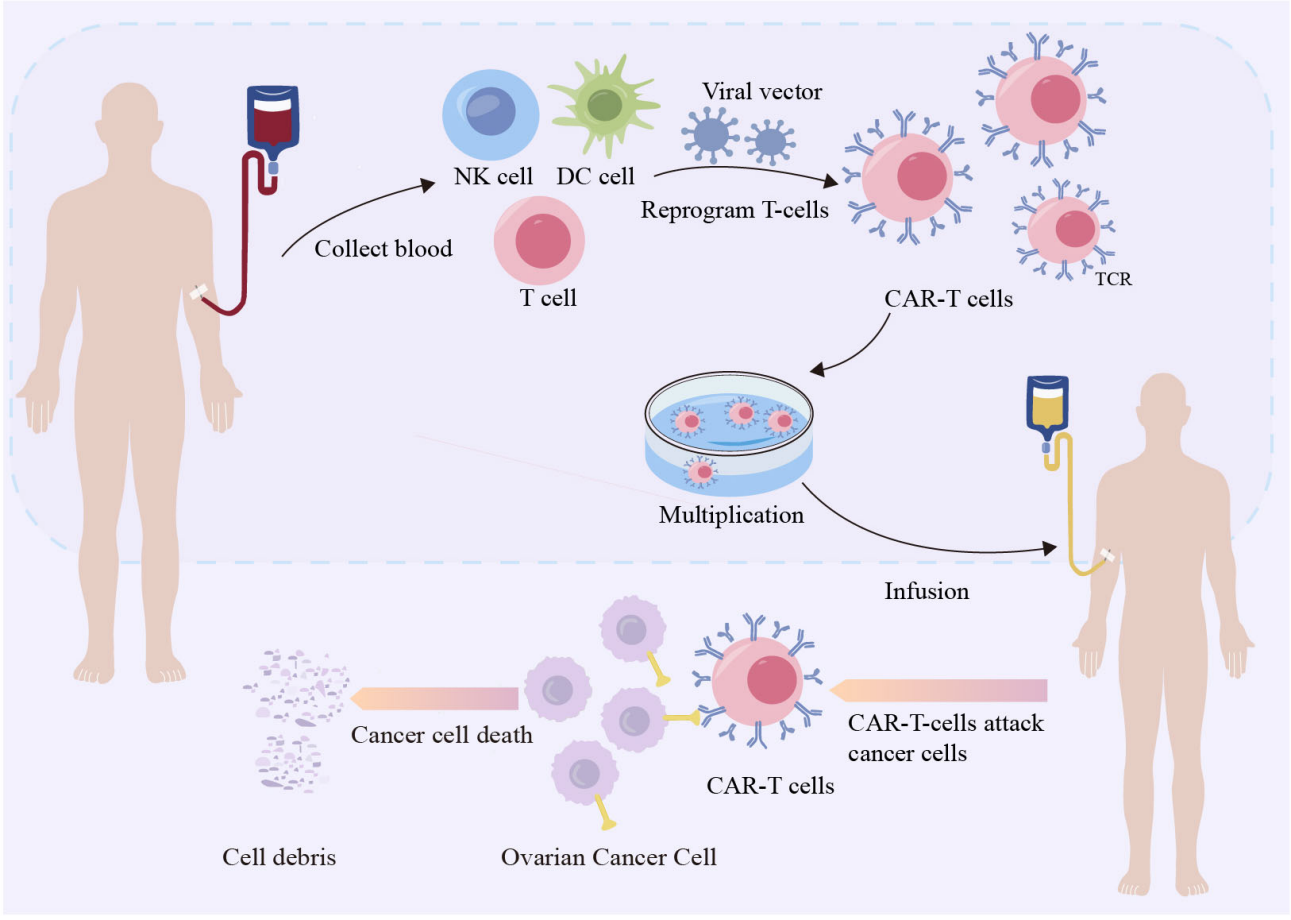
Chimeric antigen receptor-modified T cells therapy targeting ovarian cancer. Leukapheresis harvests T lymphocytes from healthy persons. Viral vectors are used to modify genes using transgenic methods. Gene-directed knock-in technology involves recombining DNA and inserting it into CARs, suicide genes, or costimulatory receptors in lymphocytes.Next, anti-CD3/anti-CD28 beads and cytokines are employed to expand T cells. The αβ-TCR antibody magnetically eliminates any remaining αβ-TCR-positive cells. Finally, the harvested T cells will be administered into the patient’s body (130).

ACT is a promising therapeutic approach that harnesses the intrinsic capabilities of the immune system to recognize and eliminate tumor cells. ACT involves isolating autologous or allogeneic immune cells, activating or genetically modifying them *in vitro*, and expanding them to produce sufficient numbers of antitumor active immune cells, which are then infused back into the patient with tumor to amplify the patient’s cellular immune function and enhance antitumor effects. ACTs include autologous lymphokine-activated killer cells, cytokine-induced killer cells (CIK), natural killer cells (NK), tumor-infiltrating lymphocytes, dendritic cells (DC), T-cell receptor-modified T cells (TCR-T), chimeric antigen receptor-modified T cells (CAR-T) (**Figure 4**), and CAR-NK cells. Recently, CAR T-cell therapy has received increasing attention as an adoptive cellular immunotherapy that targets tumors. TCR T-cell therapy for OC is still in the early stages of development (131). CAR T-cell therapy has achieved tremendous clinical success in treating patients with B-cell lymphomas and leukemia. Human epidermal growth factor receptor 2, mesothelin, MUC16, folate receptor alpha, and epithelial cell adhesion molecules are CAR targets that have been widely validated *in vitro*, and are currently under clinical investigation for the treatment of OC (132). Although previous studies have demonstrated the effectiveness of anti anti-mesothelin CAR-T treatment strategy in OC *in vitro* and *in vivo (* 133). However, targeting solid tumors has not achieved parallel success. Multiple factors may limit the efficacy and safety of this form of therapy for solid tumors, one of which is the risk of on-target, off-tumor toxicity. However, targeting bona fide cancer/ovary antigens, such as FSHR, with expression restricted to the OC (134), tumor vessels (70), and gonadal tissues (23), lowers the risk of on-target, off-tumor toxicity and makes FSHR an appealing target for T cell-based immunotherapy.

Recently, Urbanska et al. (109) developed a strategy to treat human OC by redirecting primary human T cells to target FSHR and for the first time validated the applicability of FSHR as a target for T cell immunotherapy. The findings lay the foundation for the further development of FSHR-targeted immunotherapy. In addition, T cells redirected against FSHR+ tumor cells with full-length FSH represent a promising therapeutic alternative against a broad range of ovarian malignancies with negligible toxicity, even in the presence of cognate targets in tumor-free ovaries (89). Furthermore, Bordoloi et al. (10) prepared mAbs targeting the external domain of FSHR using an FSHR vector expressed *in vivo* and identified D2 AP 11 as an effective surface-targeting mAb. Bordoloi et al. developed a bi-specific T-cell adaptor using D2 AP 11. The addition of peripheral blood mononuclear cells and T cells to a D2 AP 11 T cell engager (TCE) *in vitro* induced specific and potent killing of different genetically and immunologically escaped OC lines and reduced the tumor load in an OC-stimulated mouse model. To our knowledge, this is the first report of a bispecific TCE receptor targeting the FSHR. Moreover, the DB7.2xD2AP11 DNA-encoded bispecific NK cell engager is expressed *in vitro* and binds to Siglec-7 and FSHR, which induces potent killing in multiple ovarian tumor lines and decreases the tumor burden *in vivo* (110). These studies have described the utility of targeting FSHR in the treatment of OC. FSHR was found to have a high potency in attenuating tumor progression *in vivo* in an ovarian tumor-bearing mouse model. Notably, the potency and consistency of killing FSHR+ tumors using both the FSHR mAb and bispecific NK cell engager (NKCE)/TCE *in vitro* and *in vivo* are encouraging, providing additional tools to treat poorly responding OCs.

Generally, a cancer vaccine is defined as a vaccine targeting tumor-associated antigens and adjuvants that activate dendritic cells (DCs). The first study on the development of an OC vaccine, published in 2013, discussed the use of autologous hypochlorous acid-oxidized OC lysates to pulse DCs in a vaccine. Preclinical studies using both mice and humans were encouraging, which have led to attempts to apply it in clinical practice with positive results (135). Later research broadened the focus of the study and documented the benefits of OS rates in patients with OC using a whole-tumor lysate-pulsed DC vaccine (OCDC) in conjunction with bevacizumab and cyclophosphamide-elicited neoantigen-specific T cells. Subsequent research revealed a strong correlation between extended OS and time-to-progression rates and the addition of acetylsalicylic acid (ASA) and low-dose IL-6 to OCDC, bevacizumab, and cyclophosphamide (136). Given that many trials have demonstrated the safety and possible advantages of DC vaccination, they may have a positive impact on OC treatment results (137). In contrast, B cells and macrophages have emerged as key players in the development of innovative anticancer and DC vaccines (138). Furthermore, the Modi-1 peptide vaccine, which combines citrullinated vimentin and enolase peptides, may be beneficial for treating overconsumption in patients (139).

As FSHR expression is almost exclusive to cancer tissues and its epitopes can enhance the T cell response in OC, the idea of vaccine development has become reasonable and potentially achievable. Perales-Puchalt et al. (15) used the synthetic common sequence (SynCon) method to produce a novel DNA vaccine to overcame the immune tolerance of FSHR. The SyonCon FSHR DNA vaccine produces strong CD8+ and CD4+ cell immune responses and FSHR-redirected antibodies, delaying the progression of a highly aggressive OC model with peritoneal cancer metastasis in immunoreactive mice, and can help in OC treatment and prevent tumor recurrence after first-line treatment of FSHR+ tumors.

## 7 Challenges

Despite important advances in OC therapy, recurrent OC still presents with poor prognosis associated with a highly lethal cancer phenotype. FSHR expression in and around blood vessels in different tumors suggests that it is associated with tumor metastasis and neovascularization (140). The study of FSHR in cancer may help identify treatment and diagnostic options to improve the management of patients with cancer, as the inhibition of FSHR overexpression may be beneficial in reducing the carcinogenic effect and progression of OC (especially EOC) (89).

The FSH receptor-binding inhibitor (FRBI), an FSH antagonist, blocks FSH binding to FSHR and alters FSH activity at the receptor level. FRBI upregulates AT-rich interaction domain-containing protein 1A (ARID1A), and phosphatase and tensin (PTEN) homolog genes associated with OCs in mice (96). Furthermore, a high dose of FRBI reduced the production of c-Myc and K-Ras, which probably prevents or blocks tumorigenesis and OC progression (141). To date, little is known about the effects of FRBI on oncogenes involved in gynecological cancers in humans and animals. Therefore, the effects of FRBI on anticancer activity and progression of OC should be thoroughly investigated.

Treatments targeting FSHR are an emerging research topic in OC. Multiple studies have demonstrated that conjugated NPs combined with drugs targeting FSHR in OC display stronger antiproliferative and antitumor effects. Previous researches not only confirmed that NPs carriers could enhance the drug effects but also suggested that the FSH peptide could further enhance the antitumor activity. FSH consists of α and β chains, and some FSHR-binding domains have been identified; the affinity of peptides with FSH is FSH β33–53 > FSH β81–95 > FSH β1–15 > FSH β51–65 (14). Furthermore, a 21-amino-acid peptide, YTRDLVYGDPARPGIQGTGTF (FP21), corresponding to the FSH β33–53 sites, has the strongest binding affinity (125). Inorganic NPs have attracted considerable attention for therapeutic drug delivery because of their good biocompatibility, easy modification, design flexibility, and reduced toxicity (142). PEI is a well-known non-viral gene delivery system because of its high transfection efficiency compared to polylactic acid, poly(alkyl cyanoacrylate), and chitosan (143). Surface modification using PEG and mPEG enables PEI NPs to escape uptake by the mononuclear phagocytic system and reduces their cytotoxicity (144). However, acute toxicity observed *in vivo* limits its widespread use. The reason for this high toxicity may be that a small amount of PEG grafting leads to polyplex aggregation. Therefore, to reduce toxicity and improve the silencing efficiency of the NP complex, an FSH peptide-conjugated PEG-PEI copolymer was prepared with an increased amount of PEG grafting to silence the target gene, which promoted malignant transformation, tumor growth, and metastatic spread (125). However, the extent of the influence of the polymeric NPs has been debated. One limitation associated with the use of polymeric NPs is their limited diffusivity, which leads to inefficient distribution of drug payloads within the tumor mass (145). Therefore, a novel FSH33-targeted dendrimer-based nanocarrier was designed as a potential delivery platform for ovarian cells expressing FSHR that could also provide fertility preservation (108). In summary, a novel polymer of NPs with high selectivity for OC and normal cells expressing FSHR requires further development.

Much of the current literature on targeting FSHR in OC has focused on chemotherapeutic drugs, including PTX, which is the most commonly used drug in chemotherapy for OC. However, chemotherapeutic drugs used previously, such as PTX, target OC cells themselves rather than the entire tumor tissue (106). Thus, therapeutic approaches that specifically target cancer epithelial cells cannot completely destroy well-organized tumor tissues. Therefore, siRNA was introduced into the FSHR-mediated delivery system to silence Gro-α. The proliferation of an ovarian clear cell carcinoma cell line was inhibited after silencing Gro-α with Gro-α siRNA-loaded NPs (106). In addition, polymer- and lipid-based carriers have been used to encapsulate RNAi drugs to overcome problems such as poor stability and uptake (146). Furthermore, these data were consistent with those from *in vitro* experiments and indicated that shHK2 effectively inhibited tumor growth in cisplatin-sensitive OC (126). Therefore, an active targeting system combined with NPs is a promising tool for drug delivery. Whether and to what extent an active targeting system combined with NPs improves OC remains controversial. Further *in vivo* studies are required to investigate the therapeutic effects of this targeted complex in OC (106). Researchers have suggested that the targeting advantage of the modified NP administration system requires proper drug concentration and administration time (105). Further pharmacokinetic studies are needed to evaluate the elimination half-life, bioavailability, area under the curve, apparent volume of distribution, and long-term toxicity (105).

Ovarian tumor cells closely interact with the TME; thus, the development of treatment approaches that not only target tumor cells but also maintain their antitumor functions in this microenvironment is of particular importance (9). An increasing number of studies on immune-based therapies for OC have been performed, including treatment with ICIs, cancer vaccines, ACT, oncolytic viruses, and immunosuppression of the TME. Much of the current literature on immune-based OC therapies has focused on cancer vaccines and ACT. ACT can be classified into adoptive T-cell therapy and other immune cell types such as NK cells, CIK cells, and macrophages. Adoptive therapy with tumor-specific T-cells consists of two major forms: genetic modification of T-cells for the expression of a specific TCR and CAR (147). Recently, a different strategy for the treatment of human OC has been developed involving redirecting primary human T cells against FSHR, which validated the suitability of FSHR as a target for T cell-based immunotherapy for the first time (109). In addition, chimeric receptors using the entire FSH subunit can effectively redirect the cytotoxic activity of T cells against various patient-derived FSHR+ ovarian carcinomas (89). Chimeric receptors using entire FSH subunits may induce significant therapeutic effects in OC without detectable toxicity or alternative targeting of healthy tissues. To the best of our knowledge, this is the first report describing the generation of a vaccine against FSHR. This SynCon DNA vaccine targeting FSHR broke immune tolerance, elicited potent long-lasting CD8+ and CD4+ responses, and delayed FSHR+ tumor progression by enhancing antitumor immunity, specifically CD8+ cytotoxic T lymphocyte (CTL) responses (15). Furthermore, this approach has been extended to generate anti-FSHR mAbs and study the resulting reagents as biologics (10). To the best of our knowledge, this is the first scFv-based therapy developed for the treatment of FSHR. This could be useful for designing other scFv-based therapeutics, such as CAR-T cells, that target FSHR in OC. Researchers have summarized the current evidence of NK cell applications in the treatment of OC (148). Moreover, researchers have engineered NKCE to simultaneously engage NK cells through Siglec-7 and OC targets through FSHR, which is specific to its target cells and is potent in killing tumor cells, as evaluated using a panel of different human ovarian tumor cell lines (110).

However, immunotherapeutic strategies have shown minimal success in the treatment of OC. Immunotherapy has long been used in clinical settings to treat solid tumors; however, several difficulties hinder its advancement of cancer immunotherapy. Most immunotherapy techniques have been shown to reduce tumor progression in animal models, but are generally ineffective in patients (149). A deeper understanding of the mechanisms underlying effective anticancer responses and the various intrinsic and extrinsic variables operating on tumor cells that result in primary, adaptive, and acquired resistance to immunotherapy is necessary (150).

## 8 Conclusion

FSH and FSHR play important roles in OC cell proliferation, survival, and metastasis. NPs targeting FSHR have not only reduced side effects, but also have improved efficacy in the treatment of OC, improving OS in patients. Targeted immunotherapy against FSHR has achieved significant breakthroughs, including TCE, NKCE, and novel DNA vaccines, which can help in OC treatment and prevent tumor recurrence after the first-line treatment of FSHR+ tumors. Thus, FSHR is expected to become a new therapeutic target, bringing good news to patients with OC.
